# RAP: A Novel Approach to the Rapid and Highly Sensitive Detection of Respiratory Viruses

**DOI:** 10.3389/fbioe.2021.766411

**Published:** 2021-11-05

**Authors:** Guohao Fan, Ruiqing Zhang, Xiaozhou He, Fengyu Tian, Mingzhu Nie, Xinxin Shen, Xuejun Ma

**Affiliations:** ^1^ National Institute for Viral Disease Control and Prevention, Chinese Center for Disease Control and Prevention, Beijing, China; ^2^ Center for Biosafety Mega-Science, Chinese Academy of Sciences, Wuhan, China

**Keywords:** respiratory viruses, qPCR, RAA, highly sensitive, clinical detection

## Abstract

Recombinase aided amplification (RAA) is an emerging isothermal amplification method used for detecting various pathogens. However, RAA requires a complex and long probe to ensure high sensitivity during fluorescence assay. TaqMan probe used for quantitative PCR (qPCR) is simple and universal. Herein, we developed a new approach for detecting nucleic acids of pathogens, known as RAP (Recombinase aided PCR). The method combines RAA and qPCR to ensure a rapid and highly sensitive detection using a conventional qPCR device. RAP is a two-stage amplification process performed in a single tube within 1 hour. The method involves an RAA reaction for 10 min at 39°C (first stage) followed by 15 cycles of qPCR (second stage). Using human adenovirus 3 (HADV3) and human adenovirus 7 (HADV7) plasmids, the sensitivities of RAP assays for detecting HADV3 and HADV7 were 6 and 17 copies per reaction, respectively. The limit of RAP detection was at least 16-fold lower than the corresponding qPCR, and no-cross reaction with other respiratory viruses was observed. The results of RAP analysis revealed 100% consistency with qPCR assay. This study shows that RAP assay is a rapid, specific, and highly sensitive detection method with a potential for clinical and laboratory application.

## Introduction

Techniques for analyzing nucleic acids in samples are widely used for quantitative and qualitative detection of pathogens in clinical laboratories (Riley, 2018; Kellner et al., 2019; Pumford et al., 2020; Corman et al., 2020; Paul et al., 2020). Nucleic acid amplification is typically carried out with polymerase chain reaction (PCR) and various isothermal amplification methods (Waggoner et al., 2016), such as recombinase polymerase amplification (RPA) (Lobato and O'Sullivan, 2018), recombinase aided amplification (RAA) (Qi et al., 2019), rolling-circle amplification (RCA) (Marano et al., 2020), loop-mediated isothermal amplification (LAMP) (Zhu et al., 2020) and helicase dependent amplification (HDA) (Liu et al., 2020). PCR has the advantages of simple primers and probe design and real time fluorescence quantitative detection, however it is time-consuming and needs to be improved in the sensitivity. The isothermal amplification methods have the advantages of high sensitivity and rapidity, but the designs of multiple primers (LAMP) or long probes (RAA/RPA) are difficult (Lobato and O'Sullivan, 2018; Qi et al., 2019; Zhu et al., 2020). RAA is an emerging isothermal amplification assay used to detect pathogens at 37–42°C. RAA is highly efficient and can amplify tiny amounts of DNA fragments in 5–15 min. It uses a fluorescence probe for real-time qualitative detection. Fluorescence RAA has been successfully applied for rapid detection of various bacterial and viral pathogens and single nucleotide polymorphism (SNP) (Duan et al., 2018; Fan et al., 2019; Qi et al., 2019; Zhang et al., 2019; Chen et al., 2020; Tu et al., 2020; Wang et al., 2020). However, the length requirement of the RAA probe is 46–52 bp, which is significantly longer than the TaqMan probe. Notably, long RAA probes are difficult to design because of sequence specificity requirement. Moreover, the introduction of degenerated bases in the probe can reduce the RAA sensitivity and result in non-specific amplification. Quantitative PCR (qPCR) is the “gold standard” of nucleic acid test for many pathogens (Bonroy et al., 2007; Mahony et al., 2011; Qiu et al., 2018). The sensitivity of qPCR can be improved by nest-PCR (N-PCR). Traditional two-step N-PCR exhibit higher sensitivity but the test samples are prone to contamination. The one-tube N-PCR developed in our laboratory significantly reduced the risk of contamination but was consumed more time than qPCR (Feng et al., 2018; Zhang et al., 2020; Zhao et al., 2020). Cognizant of the strengths and weaknesses of the methods mentioned above, there is a need to integrate the strengths of the various techniques and develop next-generation technology for rapid and highly sensitive detection.

Previous research reported a dubbed Penn-RAMP, a combination of RPA and LAMP, to improve the sensitivity of RPA and LAMP alone (Song et al., 2017). In this study, we integrated the distinctive features of RAA rapidity and the probe universality of qPCR and developed a novel approach for detecting pathogens, known as RAP (Recombinase aided PCR). The principle of RAP methods involves enriching tiny amounts of target DNA fragments using RAA for 10 min then amplifying the enriched templates using qPCR with 15 thermal cycles. Furthermore, we explored the physical isolation strategy using respiratory viruses (ADV3 and ADV7) as test samples and managed to complete consecutive nucleic acid amplification and detection in a single tube within 1 hour.

## Methods

### Samples Collection

In total, 200 stock clinical samples (150 nasopharyngeal aspirate and 50 throat swabs) were collected from Hunan Provincial Center for Disease Control and Prevention and the Children Hospital of Hebei Province, China. These samples were stored at −80°C. Of note, these samples were previously tested for the presence of respiratory viruses by GeXP-based multiplex PCR/RT-PCR assay (Wang et al., 2019). The clinical samples comprised 30 human adenovirus 3 (HADV3) and 30 human adenovirus 7 (HADV7) positive samples. The rest of the samples were either positive or negative for other respiratory pathogens.

### Nucleic Acid Extraction

Total DNA and RNA were extracted from 200 μL of each clinical sample using Viral RNA/DNA Isolation Kit (Tianlong, Suzhou, China) according to the manufacturer’s instructions. The extracted nucleic acids were eluted in 50 μL of elution buffer, split into two EP tubes, and stored at −80°C C until use.

### Primer, Probe, and Plasmid Construct Design

Complete genome sequences of HADV3 and HADV7 were downloaded from the Nation Center for Biotechnology Information database (NCBI,https://www.ncbi.nlm.nih.gov/). The sequences were then aligned using VectorNTI 11.5.1 to locate their conserved regions. The RAA primers for HADV3 and HADV7 as the RAP outer primers were adopted from our previous studies (Wang et al., 2019). The respective RAP inner-primers and TaqMan probes were designed using Oligo7. These primers and probes were synthesized and purified by Shanghai Bioengineering (shanghai, China). Detailed information of the primers and probes sequences is shown in Table1.

**TABLE 1:** Primers and probes sequences used in this study.

Assay	Primer/probe	Sequence (5′- 3′)	Source
RAA	HADV3 outer-F	ATT​CCG​GCA​CAG​CTT​ACA​ATT​CAC​TCG​CTC​C	Wang et al. (2019)
	HADV3 outer-R	TCAGTAGTGG TAATGTCTTT CCCAATTTGC	
	HADV7 outer-F	ACA​ACG​GGA​GAA​GAC​AAT​GCC​ACC​ACA​TAC​AC	
	HADV7 outer-R	TCC​ATC​AAT​ATC​AGT​CCA​TGA​TTC​TTC​TCC	
qPCR	HADV3 inner-F	AGC​TTA​CAA​TTC​ACT​CGC​TCC	This paper
	HADV3 inner-R	TAG​TGG​TAA​TGT​CTT​TCC​CA	
	HADV7 inner-F	AGA​AGA​CAA​TGC​CAC​CAC​AT	
	HADV7 inner-R	CAG​TCC​ATG​ATT​CTT​CTC​C	
	HADV3 P	FAM-ACAATGCAGTAACTACCACCACAAAC-BHQ	
	HADV7 P	FAM-AAGACATTACTGCAGACAACAAGCC-BHQ	
qPCR	HADV F primer	GGYCCYAGYTTYAARCCCTAYTC	Qiu et al. (2018)
	HADV R primer	AAYTTGAGGYTCTGGYTGATCKG	
	HADV3 Probe	HEX-ACAATGCAGTAACTACCACCACAA-MGB	
	HADV7 Probe	Cy5-TTACTGCAGACAACAAGCCCAT-MGB	

**FIGURE 1:**
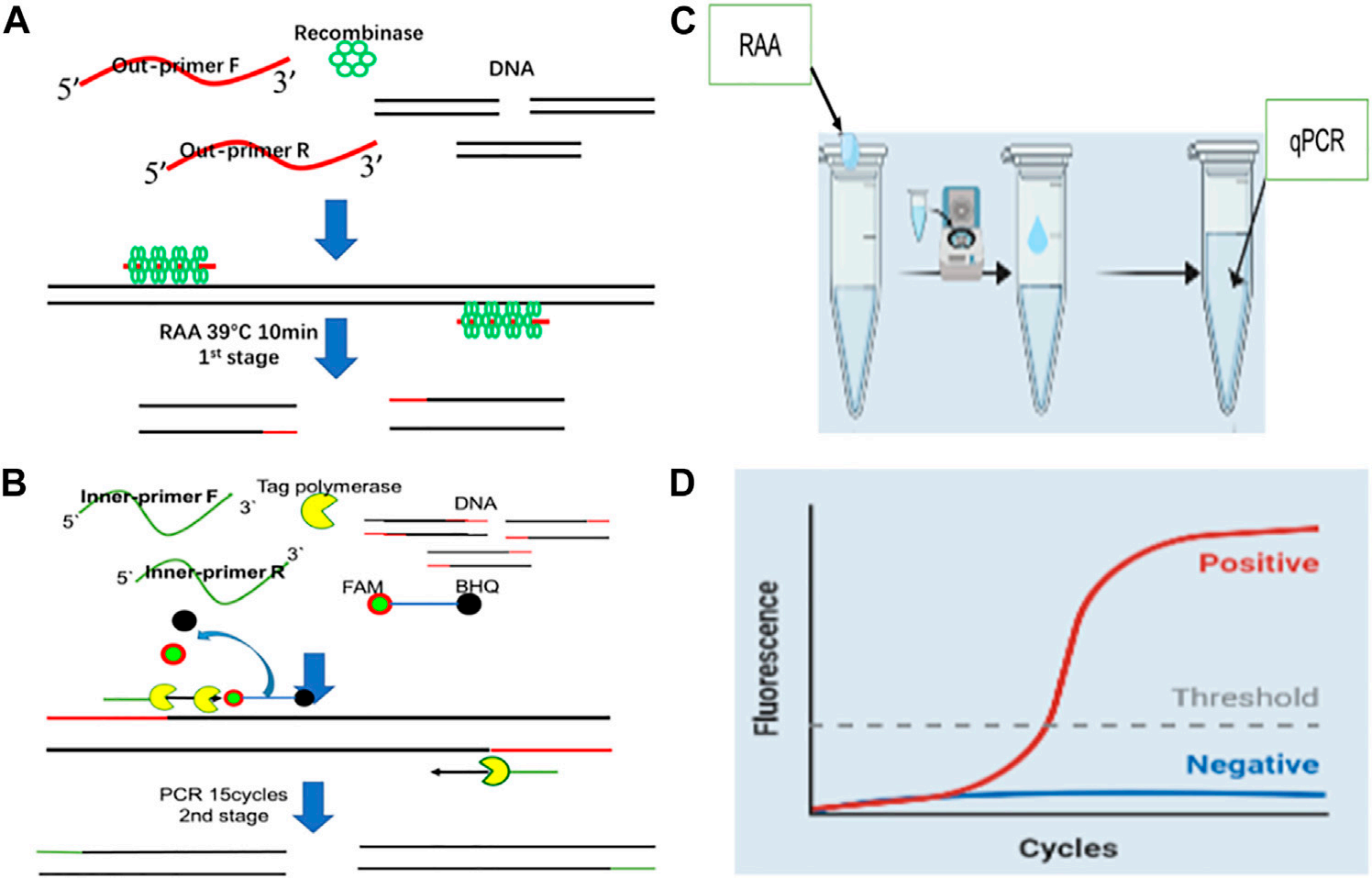
The working principle of Recombinase Aided PCR (RAP) **(A)**: RAA for 10 min at 39°C (first stage of RAP). **(B)**: qPCR 15 cycles (second stage of RAP). **(C)**: The reaction tube comprises two compartments. The tube lid (inner surface) compartment contains the RAA mix and the tube bottom compartment houses the qPCR mix. **(D)**: The results can be exhibited by real-time fluorescence curves with qPCR machine.

The target virus fragments were cloned into vector pUC57 as the plasmids. The plasmids were then quantified using Qubit™ dsDNA HS Assay Kit (Thermo Fisher Scientific, MA, United States) and transformed as copy numbers. Finally, the plasmids were diluted from 10^0^–10^9^ copies/μL using TE buffer and stored at −30°C until use. The scheme of RAP is shown in Figure 1.

### Recombinase Aided PCR Assay and Sensitivity Analysis

RAP assay was conducted in one tube and involved two stages. In the first stage, the RAA reaction was performed at 39°C in a drop suspended from the inner surface of the tube’s lid. In the second stage, qPCR was initiated at the bottom of the tube after spinning down the contents and placing the tube in a thermal cycler. The RAA reaction (total 10 μL) comprised 14 mM of magnesium ion (Mg^2+^), 200 nM of each RAA primer, 1uL of template with various concentrations and RAA reaction buffer(Jiangsu, Qitian, Bio-tech Co., Ltd..,). The qPCR (total 40 μL) contained 200 nM of each PCR primer and probe, 0.5 mM of each dNTP, and qPCR buffer (Entrans qPCR Probe Set V2, ABclonal, Wuhan, China). RAA was initially performed on the tube’s lid, but later, the tube was centrifuged to mix RAA products and qPCR mixture, then incubated at 95°C for 5 min to inactivate RAA enzyme and simultaneously begin the denaturation process. The qPCR procedure was as follows: 39°C for 10 min, 95°C for 5 min, 95°C for 15 s, 52.7°C for HADV3 or 48.9°C for HADV7 30 s and 72°C for 30 s.

The sensitivity of the RAP assay was confirmed by performing the assay using serial dilutions of the HADV3 and HADV7 plasmids from 10^0^ to 10^5^ copies/μL DNA in nuclease-free water. Meanwhile, we verified the specificity of the RAP assay using clinical samples positive for viruses other than HADV3 and HADV7.

### Optimization of Recombinase Aided PCR Assay Working Conditions

The reaction conditions for RAA and qPCR were incompatible because the working Mg^2+^ concentration was significantly different between the two assays. The suitable working concentration of Mg^2+^ for qPCR was 3 mM while the required concentration of Mg^2+^ was 14 mM in the RAA reaction mix, which was 4–5 fold higher than qPCR. As RAA could work well in a volume of 10 μL,10 μL of RAA reaction mix was then added to 30 μL or 40 μL of qPCR mix. The duration of the RAA reaction was adjusted from 5 to 12 min. The annealing temperature of qPCR was also adjusted from 45°C to 65°C and the cycle number from 10 to 30. The outcomes of these adjustments were compared and optimized. The experiments were conducted with 1 × 10^4^ copies/μL of cDNA. The amplification curve threshold time was served as the figure of merit.

### Comparing the Clinical Performance of quantitativePCR Versus Recombinase aided PCR

Among the 200 stock clinical samples tested using qPCR, 30 were positive for HADV3 and another 30 for HADV7. Subsequently, these samples were simultaneously analyzed using the RAP, and the agreement results were analyzed using SPSS statistics 26. We further tested 3 HADV3 and 3 HADV7 positive clinical samples that were 4-fold serially diluted using RAP and qPCR.

### Quantitative Detection of Recombinase aided PCR

10-fold serially diluted recombinant plasmids were simultaneously tested using qPCR and RAP. The experiment was repeated thrice. The CT values of RAP and qPCR were recorded, and the correlations between RAP and qPCR were compared using SPSS Statistics 26.

## Results

### Recombinase aided PCR Assay Optimization

**FIGURE 2:**
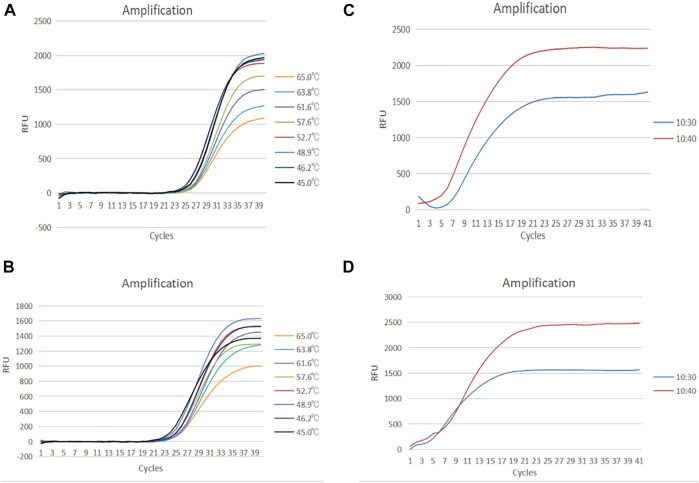
**(A)** Amplification curve of HADV3 from 45°C–65°C, **(B)** Amplification curve of HADV7 from 45°C–65°C. **(C)** Amplification curve of HADV3 with a ratio of 1 : 4 and 1:3 of RAA volume to PCR volume. **(D)** Amplification curve of HADV7 with a ratio of 1 : 4 and 1:3 of RAA volume to PCR volume.

**FIGURE 3:**
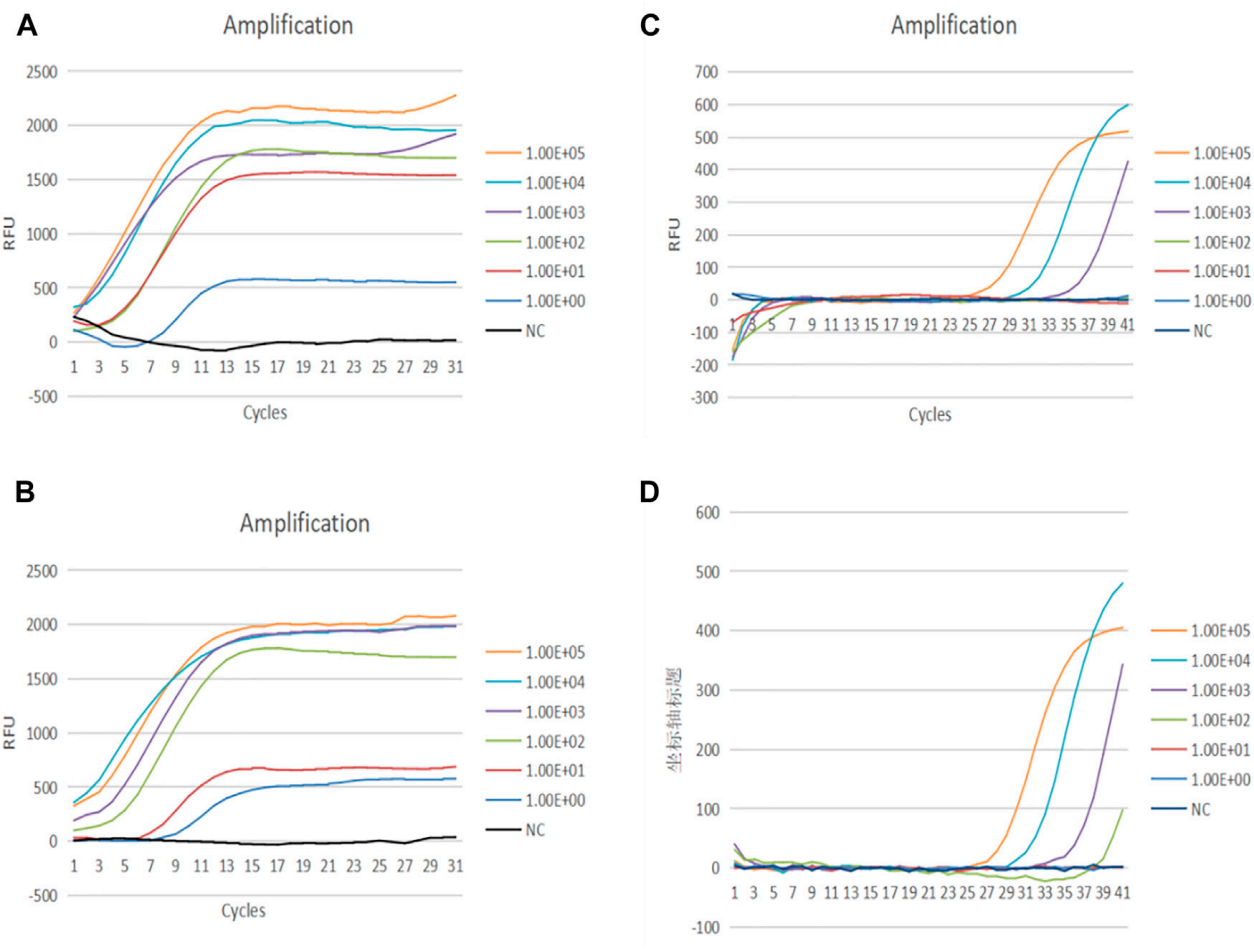
**(A)** Amplification curve of HADV3 from 100 to 105 copies/reaction using RAP. **(B)** Amplification curve of HADV7 from 10^0^–10^5^ copies/reaction using RAP. **(C)** Amplification curve of HADV3 from 10^0^–10^5^ copies/reaction using qPCR. **(D)** Amplification curve of HADV7 from 10^0^–10^5^ copies/reaction using qPCR.

The RAP performance was first assessed under different annealing temperatures of qPCR ranging from 45 to 65°C. The optimal annealing temperatures for qPCR were 52.7°C and 48.9°C for HADV3 and HADV7, respectively (Figures 2A,B). Secondly, the amplification efficiency of RAP was evaluated by comparing the volume ratio of RAA mix to qPCR mix. According to the Mg^2+^ concentration difference between RAA and qPCR mix, the ratio of 1:3 and 1:4 (10 µL RAA mix plus 30ul or 40 µL qPCR mix) were selected. The ratio of 1:4 achieved the best amplification efficiency for HADV3 and HADV7 (Figures 2C,D). The RAA assay duration was continuously adjusted from 5 to 12 min to determine the optimum time for RAA reaction in the first stage of RAP. As the time of RAA reaction increased from 5 to 10 min, the height of the fluorescence curve increased, and the CT value decreased. There was no significant difference between 8 and 10 min, but the height of the fluorescence curve reduced after 10 min (not shown). The mechanism of reduced height of the fluorescence curve after 10 min remained unclear. We suspected that some byproducts of RAA reaction might be accumulated along with the time, adversely affecting qPCR.amplification efficiency. When the different qPCR cycle numbers (10, 15, 20, 25, and 30 cycles) were compared, 15 cycles were enough to reach the plateau period in the sensitivity test for HADV3 and HADV7 (Figures 3A,B).

### Sensitivity and Specificity of Recombinase Aided PCR Assay

The sensitivity of the RAP assay for detecting HADV3 and HADV7 was determined using serially diluted recombinant plasmids. A probit analysis established the 95% limit of HADV3 and HADV7 detection using RAP as 6 and 17 copies per reaction, respectively (Table 2; Figures 3A,B). Meanwhile, the corresponding sensitivity of qPCR was between 100 and 1,000 copies/reaction (Figures 3C,D). No fluorescence signal was detected in any of the negative controls. Out of the 200 stock samples tested, 140 were positive for other respiratory viruses, including human respiratory syncytial virus (HRSV) influenza A and B viruses, rhinovirus, parainfluenza virus, human bocavirus, coronavirus, human metapneumovirus (HMPV), and other HADV species—A (HADV 31), B (HADV 14, 55), C (HADV 1, 2, 5, 6, 57), and E (HADV 4). These samples were also tested to further verify the specificity of RAP. The results of the RAP assay indicated that there is no cross-reaction with these pathogens (not shown).

**TABLE 2:** Detection limits of HADV 3 and HADV 7

Copies/reaction	No. of positive samples tested by the RAP assay for detection of HADV 3 and HADV 7	
	HADV3	HADV7
10^0^	4/8	3/8
10^1^	8/8	7/8
10^2^	8/8	8/8
10^3^	8/8	8/8
10^4^	8/8	8/8
10^5^	8/8	8/8

### Clinical Performance of Recombinase Aided PCR Versus QuantitativePCR

The 200 stock samples collected from the patients with respiratory diseases were retested using RAP. These samples were previously tested using qPCR and were found to contain 30 HADV3 positive samples (CT value from 19.2 to 28.9) and 30 ADV7 (CT value from 20.6 to 35.8) positive samples. RAP results were consistent with qPCR, indicating 100% sensitivity and specificity (Table3). Additionally, we tested three 4-fold serially diluted clinical samples with low CT values (22.0, 22.6, 26.8 for HADV3, and 26.1, 26.0, 20.6 for HADV7) using RAP and qPCR. Regarding HADV3 clinical samples, the detection limits of samples 1 and 2 reached 2^14^ times dilution at a CT value of 38.1. Meanwhile, sample 3 reached 2^10^ times dilution at a CT value of 36.6 using qPCR. The three clinical samples also tested positive for HADV3 at 2^18^ fold dilution using RAP. Hence, RAP was at least 16-fold (2^4^) sensitive than qPCR. Regarding HADV7 clinical samples, RAP was also at least 16-fold sensitive than qPCR (Supplementary Table S4).

**TABLE 3:** Detection of HADV3 and HADV7 in clinical samples.

Virus	qPCR		RAP		Agreement
	Positive	Negative	Positive	Negative	
HADV3	30	170	30	170	100.00%
HADV7	30	170	30	170	100.00%

### Quantitative Analysis of Recombinase Aided PCR

Serially diluted 10^0^–10^5^ copies of plasmids were tested to formulate the corresponding quantitative curves of RAP. Based on the results, the curve started to rise at the beginning of qPCR when the initial template concentration was above 10^3^ copies. However, no Ct value was generated at this point, suggesting that a very high concentration of template was accumulated after a 10 min RAA reaction. In addition, there was no correlation between the plasmids of different concentrations and the corresponding CT values. Therefore, we speculated that the amplification efficiency of RAA was high enough even when the amount of template was low and RAA reaction occurred randomly, resulting in the fluctuation of CT values (Supplementary Table S5).

## Discussion and Outlook

Respiratory viruses, such as HADV3 and HADV7 cause acute respiratory tract infection (ARTIs), presenting as fever, cough, and pneumonia. These conditions can lead to death in severe cases (Mahony, 2008; Rebelo-de-Andrade et al., 2010; Tregoning and Schwarze, 2010; Mahony et al., 2011; To et al., 2017). Various methods have been applied to detect these viruses, including isothermal nucleic acid amplification and PCR-based amplification technique (Zhao et al., 2015; Lim et al., 2018; Pumford et al., 2020; El-Tholoth et al., 2021; Ganguli et al., 2021; Zhang et al., 2021). RAA can accomplish sample detection in 30 min with a sensitivity of a single copy; however, the RAA probe is too long (46–52bp) and, therefore, difficult to design. The detection limit of qPCR assay is 100 copies/reaction for both HADV3 and HADV7^[36]^, which is too low for clinical sample test. N-PCR exhibits the same high sensitivity as RAA but is time consuming (takes more than 2 h) (Feng et al., 2018; Zhao et al., 2020).

In this study, we established a novel N-PCR approach, named RAP (recombinase aided PCR) by integrating the merits of RAA and qPCR detection techniques. RAP is a robust detection method that is more rapid and sensitive than the conventional qPCR. The principle of RAP involves a first-round amplification step using one pair of RAA primers (outer primers) followed by a second round of amplification using one pair of qPCR primers (inner primers) and a qPCR probe. The whole process is performed in a single tube. The RAA products are placed in the lid of the tube and briefly centrifuged to mix with the qPCR buffer at the bottom of the tube. The qPCR occurs at the bottom of the tube after heat activation of a heat-start Taq DNA polymerase. Meanwhile, the RAA components get inactivated at this point. RAP capitalizes on the rapid and high amplification efficiency of RAA and the flexibility of TaqMan probe of qPCR to detect pathogens. It avoids the design of a complex RAA probe. RAP assay for respiratory DNA viruses (HADV3 and HADV7) demonstrated detection limits of 6 and 17.0copies per reaction for HADV3 and HADV7, respectively (probit analysis *p* ≤ 0.05), which are lower than those of corresponding qPCR. The results of clinical application of RAP in testing 200 samples were consistent with those of qPCR. Of note, no cross-reaction was observed with other common respiratory virus-positive samples. The testing results using serially diluted clinical samples also indicated that RAP possesses higher sensitivity. The rapidity of RAP (1 h and single-tube assay) protects the reaction from contaminating and saves running time compared to qPCR. RAP does not depend on the isothermal detection device since it can be performed on a conventional real-time thermal cycler, indicating that it can be applied in general laboratories. Overlapping of qPCR primers with RAA primers was explored in our preliminary study to further simplify the design of RAP primers. And no adverse impact on the RAP amplification efficiency was observed.

However, RAP has some limitations. Although the RAP analysis result is shown by CT value of qPCR, the CT value cannot quantify the virus titer in the original samples. Because of the extremely high amplification efficiency of RAA, most respiratory clinical samples generate no or very low CT values in the second stage of the qPCR, suggesting that the amplification for most samples reaches the plateau phase after RAA. Further improvement of RAP will aim to 1) use a Taq enzyme that can function in the RAA reaction mix containing a high Mg^2+^ concentration to allow the use of only one mix in the tube. 2) develop multiple detections of pathogens. 3) reduce RAA reaction time and increase qPCR cycles for quantitative detection of pathogens.

Despite the few limitations of RAP in this proof-of-concept study, RAP can serve as a robust, rapid, and highly sensitive detection platform with a wide potential application in the detection of DNA/RNA viruses, bacteria, and SNPs. In particular, RAP can be used for fast screening of newly infected asymptomatic patients, sensitive detection of cerebrospinal fluid samples with low viral load, monitoring low frequency of drug resistance pathogens, such as *Mycobacterium tuberculosis,* and monitoring the treatment efficacy of HIV patients with low viral load.

Our study demonstrated that the RAP method enables a rapid and highly sensitive detection using a conventional qPCR device. The merits of RAP are the high sensitivity and adaptability, making RAP a great potential to be cooperated with existing qPCR and microfluidic devices. Further attempts will be made to integrate RAP with microfluidic technology and develop a biosensor-based instrument towards clinical and point-of-care applications in the rapid, field detection of multiplex pathogens in the future.

## Data Availability

The original contributions presented in the study are included in the article/Supplementary Material, further inquiries can be directed to the corresponding authors.
