# Supplementary material for: RAP: A Novel Approach to the Rapid and Highly Sensitive Detection of Respiratory Viruses
Source: Front Bioeng Biotechnol. 2021 Nov 5;9:766411. doi: 10.3389/fbioe.2021.766411 (PMC8602363; doi:10.3389/fbioe.2021.766411)
Supplement: Supplementary file 1 [file Table1.docx]

Supplementary table 1. Detection of HADV3 and HADV7 using serially diluted clinical samples

|  | Results of qPCR | | | | | | | | | | | | | | | | | | | | | |  | | Results of RAP | | | | | | | | | | | | | | | | | | | | | |  |  |
| --- | --- | --- | --- | --- | --- | --- | --- | --- | --- | --- | --- | --- | --- | --- | --- | --- | --- | --- | --- | --- | --- | --- | --- | --- | --- | --- | --- | --- | --- | --- | --- | --- | --- | --- | --- | --- | --- | --- | --- | --- | --- | --- | --- | --- | --- | --- | --- | --- |
|  | 2^0^ | | 2^2^ | | 2^4^ | | 2^6^ | | 2^8^ | | 2^10^ | | 2^12^ | | 2^14^ | | 2^16^ | | 2^18^ | | Negative control | |  | | 2^0^ | | 2^2^ | | 2^4^ | | 2^6^ | | 2^8^ | | 2^10^ | | 2^12^ | | 2^14^ | | 2^16^ | | 2^18^ | | Negative control | | |  |
| HADV3 | | 22.0 | | 24.8 | | 26.9 | | 29.3 | | 31.8 | | 36.6 | | 37.1 | | 38.1 | | —^a^ | | — | | — | |  | | +^b^ | | + | | + | | + | | + | | + | | + | | + | | + | | + | | - | | |
|  |  | 22.6 | | 25 | | 27.2 | | 28 | | 32.1 | | 35.2 | | 37.4 | | 38.1 | | — | | — | | — | |  | | + | | + | | + | | + | | + | | + | | + | | + | | + | | + | | - | | |
|  |  | 26.8 | | 29 | | 31.5 | | 33.7 | | 36.6 | | — | | — | | — | | — | | — | | — | |  | | + | | + | | + | | + | | + | | + | | + | | + | | + | | + | | - | | |
| HADV7 | | 26.1 | | 28.1 | | 31.5 | | 34.0 | | — | | — | | — | | — | | — | | — | | — | |  | | + | | + | | + | | + | | + | | + | | + | | + | | + | | + | | - | | |
|  |  | 26.0 | | 28.3 | | 30.5 | | 33.1 | | 35.6 | | 36.0 | | — | | — | | — | | — | | — | |  | | + | | + | | + | | + | | + | | + | | + | | + | | + | | + | | - | | |
|  |  | 20.6 | | 22.9 | | 25.2 | | 27.4 | | 29.6 | | 32.2 | | 34.1 | | 36.1 | | — | | — | | — | |  | | + | | + | | + | | + | | + | | + | | + | | + | | + | | + | | - | | |

a:-, negative. b:+, positive.
