# Supplementary material for: RAP: A Novel Approach to the Rapid and Highly Sensitive Detection of Respiratory Viruses
Source: Front Bioeng Biotechnol. 2021 Nov 5;9:766411. doi: 10.3389/fbioe.2021.766411 (PMC8602363; doi:10.3389/fbioe.2021.766411)
Supplement: Supplementary file 2 [file Table2.docx]

Supplementary table 2. Results of HADV3 and HADV7 using RAP

| Copies/reaction | CT of HADV3 | | | CT of HADV7 | | |
| --- | --- | --- | --- | --- | --- | --- |
| 10^0^ | 12.45 | —^a^ | 10.05 | — | 9.86 | — |
| 10^1^ | 8.49 | 7.83 | 9.44 | 6.97 | 7.53 | 3.34 |
| 10^2^ | 2.30 | 3.15 | NA | NA | 3.96 | 4.01 |
| 10^3^ | NA^b^ | NA | 2.95 | NA | NA | NA |
| 10^4^ | NA | NA | NA | NA | NA | NA |
| 10^5^ | NA | NA | NA | NA | NA | NA |

a:—, negative, b: NA, positive but no CT value
